# Emergence of probabilistic representation in the neural network of primary visual cortex

**DOI:** 10.1016/j.isci.2022.103975

**Published:** 2022-02-26

**Authors:** Ang A. Li, Fengchao Wang, Si Wu, Xiaohui Zhang

**Affiliations:** 1 Academy for Advanced Interdisciplinary Studies, IDG/McGovern Institute for Brain Research, Peking-Tsinghua Center for Life Sciences, Beijing, China; 2 School of Psychology and Cognitive Sciences, Peking University, Beijing, China; 3 State Key Laboratory of Cognitive Neuroscience and Learning, IDG/McGovern Institute for Brain Research, Beijing Normal University, Beijing, China

**Keywords:** Bioinformatics, Cellular neuroscience, Neural networks, Sensory neuroscience

## Abstract

During the early development of the mammalian visual system, the distribution of neuronal preferred orientations in the primary visual cortex (V1) gradually shifts to match major orientation features of the environment, achieving its optimal representation. By combining computational modeling and electrophysiological recording, we provide a circuit plasticity mechanism that underlies the developmental emergence of such matched representation in the visual cortical network. Specifically, in a canonical circuit of densely-interconnected pyramidal cells and inhibitory parvalbumin-expressing (PV+) fast-spiking interneurons in V1 layer 2/3, our model successfully simulates the experimental observations and further reveals that the nonuniform inhibition plays a key role in shaping the network representation through spike timing-dependent plasticity. The experimental results suggest that PV + interneurons in V1 are capable of providing nonuniform inhibition shortly after vision onset. Our study elucidates a circuit mechanism for acquisition of prior knowledge of environment for optimal inference in sensory neural systems

## Highlights

• Computational and experimental methods are combined to representation in mice V1
• Nonuniform inhibition plays a key role in shaping the network representation
• PV + interneurons provide nonuniform inhibition shortly after vision onset

## Graphical abstract

**Figure:**
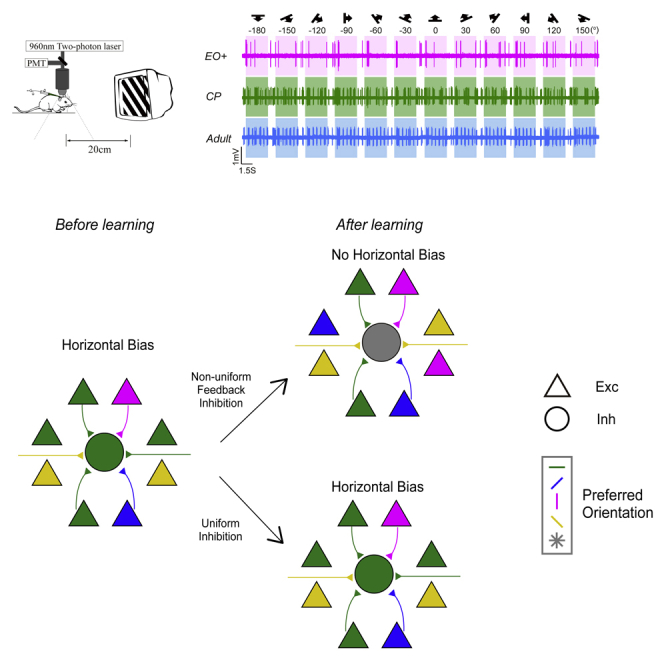

## Introduction

Neural networks of animals and humans develop probabilistic representations that match the environment and guide behaviors. In the mammalian primary visual cortex (V1), neurons encode oriented bars or edges of light in their receptive fields (Hubel and Wiesel, 1959). Collectively, the neural population represents the statistics of the visual environment (Pouget et al., 2000). A recent framework for probabilistic representation in the sensory cortex proposes that the prior distribution of stimuli can be represented by the distribution of preferred orientation in the neural population (Fischer and Peña, 2011; Girshick et al., 2011; Ganguli and Simoncelli, 2014). Indeed, this proposal is supported by experimental evidence: under normal conditions, neurons in the V1 of adult animals and humans show an overrepresentation of the cardinal orientations, which coincides with the local orientation distribution measured in photographs (Chapman and Bonhoeffer, 1998; Furmanski and Engel, 2000; Girshick et al., 2011; Hagihara et al., 2015), whereas an altered environment — the restriction of visual experience to contours of only one orientation (stripe-rearing) — leads to an overrepresentation of the experienced orientations (Stryker et al., 1978; Kreile et al., 2011). However, how such probabilistic representation emerges during the development of the cortical network is not known (Seriès and Seitz, 2013).

We aimed to address this question by examining the development of a canonical microcircuit that has been proposed as the building block for predictive coding (Bastos et al., 2012) and Bayesian inference (Darlington et al., 2018; Nessler et al., 2013). This microcircuit consists of densely interconnected populations of excitatory and inhibitory neurons (Hofer et al., 2011): previous experiments suggested that the connectivity between pyramidal cells (PCs) and inhibitory parvalbumin-expressing (PV+) interneurons in cortical layer 2/3 are random, reciprocal, and dense, with connection probability reaching 50% (Miao et al., 2016; Avermann et al., 2012). This microcircuit is largely mature early before eye-opening (EO) and shapes the entire process of visual development (Magueresse and Monyer, 2013), including the sharpening of orientation tuning and the onset of a critical period (CP) of experience-dependent neuronal connection refinements during early postnatal development (Lee et al., 2012; Kuhlman et al., 2013).

In computational studies, this microcircuit is frequently modeled as a feedback inhibition network (Avermann et al., 2012). Combined with a synaptic learning rule of spike timing-dependent plasticity (STDP), it has been used to study the development of V1, including the emergence and sharpening of orientation tuning (Song and Abbott, 2001; Sadeh et al., 2015). We propose that this feedback inhibition network is ideal for probabilistic representation. Unlike winner-take-all (WTA) inhibition (Rumelhart and Zipser, 1985), where a single PC firing can suppress firing of other PCs in the network, feedback inhibition provides a softer form of inhibition (Avermann et al., 2012; Jonke et al., 2017), where the level of inhibition is proportional to the level of excitation (Isaacson and Scanziani, 2011). This softer form of inhibition allows a subset of excitatory neurons to respond to a specific input feature, which enables the neural population to represent a probability distribution.

To study the emergence of probabilistic representation in the cortical microcircuit, we combined mathematical modeling with electrophysiological experiments, emphasizing the role of PV + inhibitory neurons — the major player in feedback inhibition. Based on the feedback inhibition network model with STDP, we built a computational model that mimics the development of cortical microcircuits under normal and altered environments. In addition, we performed *in vivo* electrophysiological recordings to measure the tuning properties of PCs and inhibitory PV + interneurons in mouse V1 across different developmental stages: shortly after EO (EO+), during the CP, and the adult stage. Our modeling and experimental results showed that the distributions of preferred orientations of PCs and inhibitory PV + interneurons are tightly matched across different developmental stages. Furthermore, an ablation study in the network model suggested that feedback inhibition provided by PV + interneurons is necessary for the emergence of probabilistic representation in the microcircuit. In summary, our work delineates a crucial role of feedback inhibition in the developmental emergence of probabilistic representation in the cortical network.

## Results

### Emergent properties of the feedback inhibition network

We studied the emergence of probabilistic representation in the cortical network using a ubiquitous microcircuit model, referred to as the feedback inhibition network (Grossberg, 1976; Jonke et al., 2017). The network mimics the densely interconnected excitatory and inhibitory neurons in the cortex. Previous experiments have shown that the connectivity between PCs and PV + interneurons in layer ⅔ is random, reciprocal, and dense — with a connection probability reaching 50% (Miao et al., 2016; Avermann et al., 2012). This microcircuit matures early before EO and shapes the entire process of visual development including the sharpening of orientation tuning and the onset of the CP (Lee et al., 2012; Magueresse and Monyer, 2013; Kuhlman et al., 2013). Moreover, this microcircuit has been proposed as the building block for predictive coding (Bastos et al., 2012) and Bayesian inference (Darlington et al., 2018; Nessler et al., 2013).

**Figure 1:**
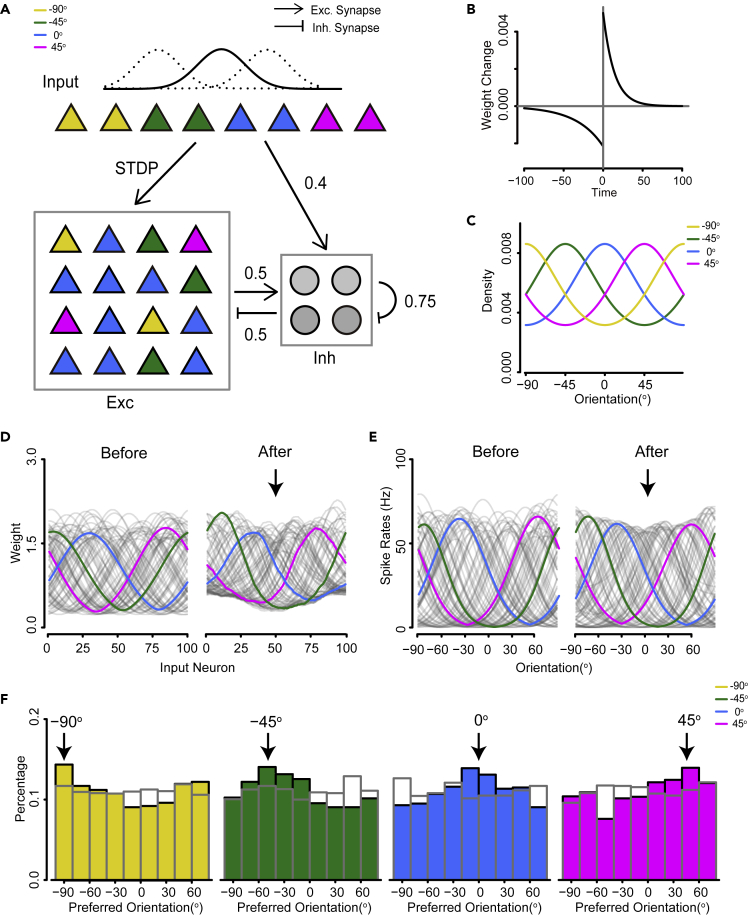
Emergent properties of the cortical network under the stripe-rearing condition (A and B) Schematic of the network model structure. Input neurons encode an orientation variable with a Gaussian profile of their firing rates. Triangles represent an excitatory population, whereas circles represent an inhibitory population. Colors represent neurons with different preferred stimuli. Numbers on the arrows represent connection probabilities between populations (B) The standard STDP learning rule used in the model, which specifies the change of synaptic weight for a pre-post paring with a time difference $\Delta t=t_{post}-t_{pre}$ between a postsynaptic spike at time $t_{post}$ and a presynaptic spike at time $t_{pre}$. (C) Prior probabilities of orientations under different stripe-rearing conditions. Yellow, green, blue, and magenta lines indicate experienced orientation at −90°, −45°, 0°, and 45°. (D) Feedforward connection weights of simulated excitatory neurons before and after training under the stripe-rearing condition at 0° (smoothed by a moving-average filter with 10 nearest points). Three sample neurons are highlighted in magenta, green, and blue. (E) Simulated excitatory neurons before and after training under stripe-rearing conditions at 0°. The same neurons as in D are highlighted in magenta, green, and blue. (F) Histograms of preferred orientations under each rearing condition, experienced orientations are specified above the graphs. Gray bars represent initial conditions.

The model consisted of interconnected excitatory and inhibitory neural populations, of which 80% were excitatory neurons and 20% inhibitory (Figure 1A). The inhibitory neurons were recurrently connected. The connection probabilities between different types of neurons are in accordance with the experiment data from the visual cortex (Miao et al., 2016). Each neuron was modeled as a leaky integrate-fire neuron. Both excitatory and inhibitory neurons received feedforward inputs modeled as Poisson spikes. But only the feedforward connections received by excitatory neurons were plastic and subject to a standard STDP rule (Figure 1B), with homeostatic weight rescaling. Similar models have been used to study visual development (Song and Abbott, 2001; Sadeh et al., 2015; Jonke et al., 2017), but how probabilistic representation emerges in the network is not well understood.

We examined the emergent properties of the feedback inhibition network in a setting that mimicked the stripe-rearing condition: the restriction of visual experience to contours of only one orientation. Previous experiments have shown that stripe-rearing leads to an overrepresentation of the experienced orientation among neurons in the visual cortex (Kreile et al., 2011; Stryker et al., 1978). In our simulation, the orientation input was encoded by Poisson spiking neurons with a Gaussian profile of firing rates (Song and Abbott, 2001; Sadeh et al., 2015). Before training, the excitatory neurons were initialized to be orientation-selective, with uniformly distributed preferred orientations (Figure 1F, gray bar). To mimic the stripe-rearing condition, the prior distribution of orientation inputs followed a von-Mises distribution that peaked at the experienced orientation (Figure 1C). We selected 4 stripe-rearing conditions with experienced orientations at −90°, −45°, 0°, and 45°(Kreile et al., 2011).

Stripe-rearing had a strong effect on simulated neurons. After training, the feedforward connection weights of simulated neurons tended to shift toward the experienced orientation (Figure 1D). As a result, the tuning curves also shifted toward this orientation (Figure 1E). On the population level, the distribution of preferred orientations showed an overrepresentation at the experienced orientation (Figure 1F), consistent with the experimental observation. To quantify the specific effect because of stripe-rearing, we compared the increase in the fraction of neurons preferring the experienced orientation to the decrease in preference for the orthogonal orientation (Kreile et al., 2011). The average specific effect of stripe rearing was 12.68% (−90°: 10.65±8.55%, −45°: 13.95±8.01%, 0°: 13.05±5.13%, and 45°: 13.05±6.39%). Our results suggest that the distribution of preferred stimuli in the feedback inhibition network shifts according to the prior distribution of the input, leading to the stripe-rearing effect.

### Emergence of the overrepresentation of cardinal orientations in the cortical network

**Figure 2:**
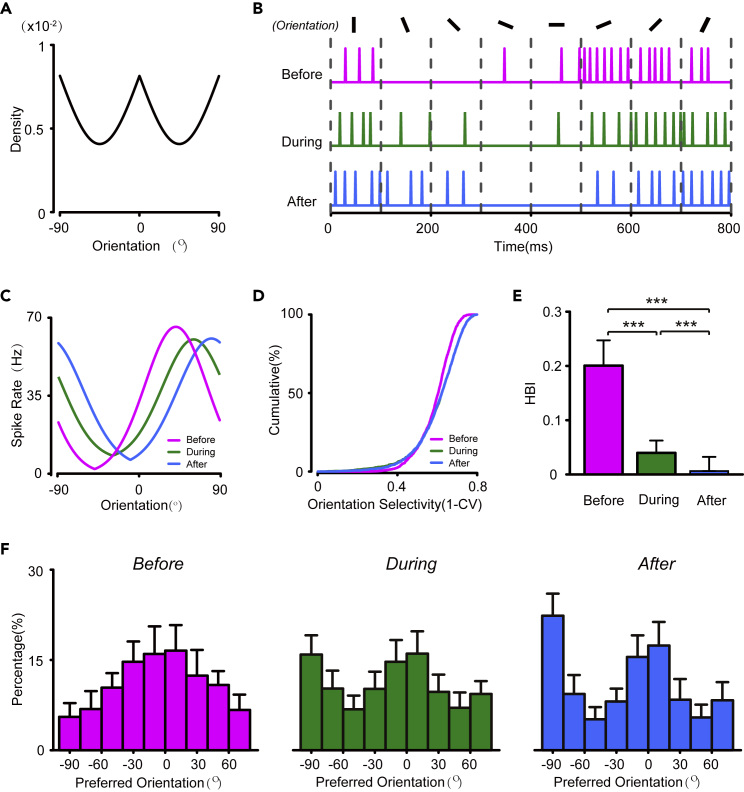
Emergence of the over-representation of cardinal orientations in the feedback inhibition model (A). Emergence of the overrepresentation of cardinal orientations in the feedback inhibition model (A) Prior probabilities of the orientations in natural images (B) Spike trains of a representative simulated excitatory neuron in response to stimuli with eight different orientations, before (magenta), during (green), and after (blue) learning. (C) Orientation tuning curves of a simulated excitatory neuron at different learning stages (the same neuron as in B). (D) Distribution of the orientation-selective index (OSI) of simulated excitatory neurons at different learning stages. (E) Horizontal bias index (HBI) of simulated excitatory neurons at different learning stages. (F) Distribution of preferred orientation of simulated excitatory neurons before (left), during (middle), and after (right) learning. ∗p < 0.05,∗∗p < 0.01, ∗∗∗p < 0.001, Kolmogorov–Smirnov test in D and t-test in (E) Data are represented as mean ± SEM.

During visual development, neurons in V1 develop an overrepresentation of cardinal orientations (Hagihara et al., 2015), which coincides with the local orientation distribution measured in photographs (Girshick et al., 2011) (Figure 2A). This process can be understood as the neural network matching its internal model to the environment for optimal inference (Girshick et al., 2011; Fischer and Peña, 2011; Ganguli and Simoncelli, 2014). Here, we combined computational and experimental methods to study its circuit mechanism.

We studied the emergent properties of the feedback inhibition network under a condition that mimicked visual development. Experiments showed that, in mice shortly after EO, the distribution of preferred orientation in PCs had a horizontal bias, with more neurons preferring horizontal orientations (Hagihara et al., 2015). Thus, we initialized the preferred orientations of the excitatory population with a von-Mises distribution that peaks at horizontal orientation (Figure 2F left). During learning, orientation inputs were randomly drawn from a prior distribution, mimicking the local orientations measured in natural images (Girshick et al., 2011; Wei and Stocker, 2015) (Figure 2A). The tuning properties of neurons were tested by 8 different orientation stimuli before, during, and after the learning period (Figure 2B).

We found that an overrepresentation of the cardinal orientation emerged in the network model. During learning, the preferred orientation of excitatory neurons tended to shift toward the vertical orientation, which was initially underrepresented by the network. On the population level, the horizontal bias in the distribution of preferred orientations of excitatory neurons became cardinal, with peaks at both horizontal and vertical orientations (Figure 2F), and this was stable during the latter half of the training period. To assess these changes, we quantified the horizontal bias using the horizontal bias index (HBI) (Hagihara et al., 2015). The excitatory neurons before learning had a significantly larger HBI than those during and after learning (Figure 2E, t-test between before and during learning, p < 2.2E-16; between before and after learning, p < 2.2E-16). However, the distribution of the orientation selectivity index (OSI) does not change significantly during training (Figure 2D), suggesting that the overrepresentation of cardinal orientation is solely caused by a shift of preferred orientation of excitatory neurons. Our model successfully reproduced the emergence of the overrepresentation of cardinal orientation during visual development.

**Figure 3:**
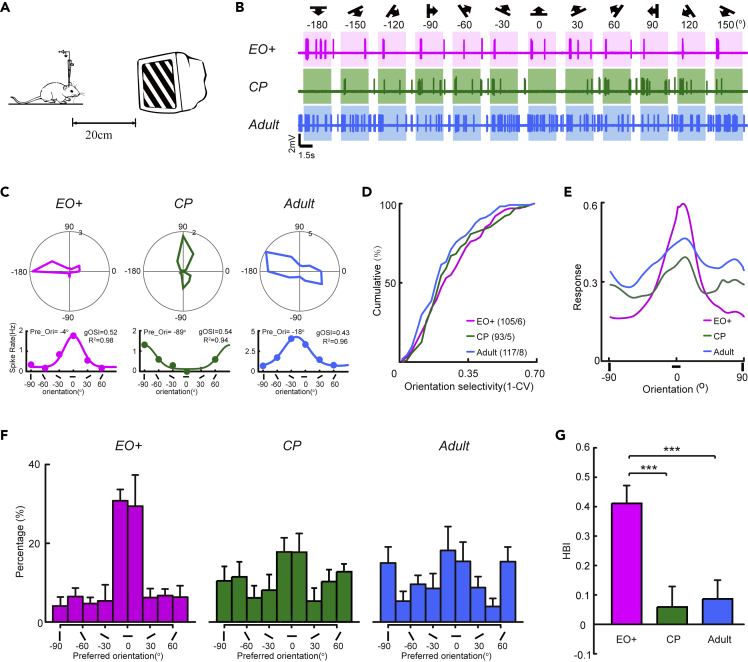
Emergence of the overrepresentation of cardinal orientations in the cortical network of V1 during development (A) Schematic illustrating *in vivo* single-unit recording of spike responses from layer 2/3 PCs in mouse V1. (B) The responses of representative neurons at different postnatal stages to drifting gratings with 12 different directions. The stimulus directions and orientations are indicated by the symbols shown above. (C and D) Upper, the direction-tuned responses of representative neurons in (A) Lower, curves denoting the method to use one-peak Gaussian function fitting to detect the preferred orientation of representative neurons in (B). (D) Cumulative distributions of OSI values of PCs at three postnatal stages. (E) Population tuning curves of PCs at three postnatal stages. (F) Distributions of preferred orientations of PCs in EO + mice (98 cells / 6 mice), CP mice (89 cells / 5 mice), and Adult mice (113 cells/ 8 mice). (G) HBI of PCs in 3 postnatal stages. ∗p < 0.05,∗∗p < 0.01, ∗∗∗p < 0.001, Kolmogorov–Smirnov test in D and t-test in (G) Magenta, EO+; green, CP; blue, Adult. Data are represented as mean ± SEM.

To validate our model results, we used *in vivo* extracellular single-unit recording to measure the orientation selectivity of PCs in the binocular area of the mouse V1 at different developmental stages: shortly after EO (EO+, postnatal day 17–18, P17–18), during the CP (P27-28), and in the adult stage (P55-56). For each stage, we measured the spiking activity of putative PCs in response to drifting grating stimuli with 12 different directions (6 orientations) (Figures 3A and 3B). We computed the preferred orientation of each PC by fitting the spike-rate vs orientation tuning curve with a Gaussian function and calculate the orientation selective index (OSI) to measure their orientation tuning amplitudes (Figure 3C). We found that for PCs recorded shortly after EO, the OSI amplitudes were already comparable to those during the CP (two-sample Kolmogorov– Smirnov test between P17-P18 and P27-28, p = 0.557) and the later adult stage (two-sample Kolmogorov– Smirnov test between P17-P18 and P55-56, p = 0.053) (Figure 3D), confirming the previous findings that the orientation selectivity of PCs largely matures around EO (Hagihara et al., 2015; Hoy and Niell, 2015).

At the population level, we analyzed the distribution of preferred orientations of putative PCs at three developmental stages. The distribution showed an apparent bias to the horizontal orientation in those mice examined shortly after EO, consistent with previous findings (Hagihara et al., 2015). However, during the CP the distribution evolved to become bimodal with two peaks at the horizontal and vertical orientations, and this further remained stable through the adult stage (Figure 3F). The horizontal biases were quantified with the HBI, which showed that the PCs shortly after EO had a significantly stronger bias toward the horizontal orientation than those during the CP (t-test, p = 1.75E-04) and the adult stage (t-test, p = 3.22E-04, Figure 3G). Moreover, we also computed the population tuning curve for the recorded PC population by averaging the normalized tuning curves of individual PCs (McAdams and Maunsell, 1999). The population tuning curve quantitatively reflects the population activity to different orientations; it clearly showed a bias to the horizontal orientation shortly after EO, whereas the bias was no longer evident during the CP and in the adult (Figure 3E). This developmental change in the population tuning curve of PCs agreed with the observed developmental evolution of the distribution of preferred orientations. Thus, these experimental recordings directly reveal a switching process from the overrepresentation of horizontal orientation to the cardinal orientations in V1 layer 2/3 PCs during a period before the CP, and these results verify the model simulations.

### Inhibitory neurons provide nonuniform feedback inhibition during early development

Inhibitory neurons play a key role in visual development (Espinosa and Stryker, 2012; Magueresse and Monyer, 2013), but whether and how they shape the overrepresentation of cardinal orientations are not known. In this section, we consider the changes in tuning properties of inhibitory PV + interneurons during visual development.

**Figure 4:**
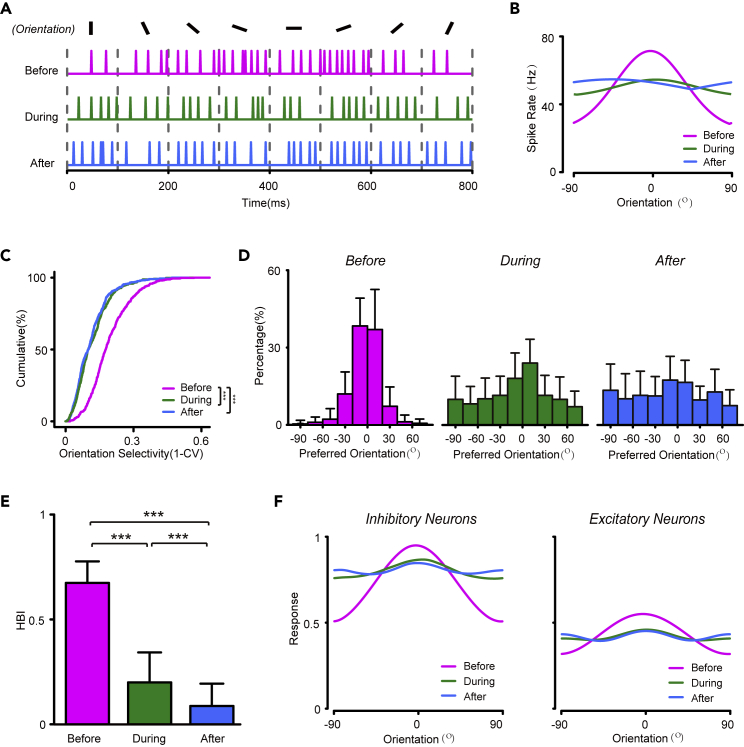
Simulated inhibitory neurons provide competitive inhibition during learning (A). Simulated inhibitory neurons provide competitive inhibition during learning (A) Spike trains of a representative simulated inhibitory neuron to stimuli with 8 different orientations, before (magenta), during (green), and after (blue) learning (B) Orientation tuning curves of a representative inhibitory neuron at the different learning stages (the same neuron as in A). (C) Distribution of the orientation selective index (OSI) of simulated inhibitory neurons at the different learning stages. (D) Distribution of preferred orientation of simulated inhibitory neurons before (left), during (middle), and after (right) learning. (E) The horizontal bias index (HBI) of simulated inhibitory neurons at the different learning stages. (F) Population tuning curves of simulated excitatory (left) and inhibitory (right) populations at different learning stages. ∗p < 0.05,∗∗p < 0.01, ∗∗∗p < 0.001, Kolmogorov–Smirnov test in C and t-test in (E) Magenta, EO+; green, CP; blue, Adult. Data are represented as mean ± SEM.

In the network model, we found structural changes in the tuning properties of inhibitory neurons during learning. First, the inhibitory neurons showed a modest level of orientation tuning before learning, and this was lost during learning (Figures 4A and 4B). At the population level, the OSI significantly decreased during learning (Figure 4C, two-sample Kolmogorov–Smirnov test, p <2.2E-16). Because PV + inhibitory neurons receive dense and unselective connections from the excitatory population, changes in their orientation selectivity may reflect changes in the distribution of preferred orientations as well as the population tuning curves of excitatory neurons (Runyan and Sur, 2013).

Second, inhibitory neurons provided nonuniform inhibition during the early learning stage, most prominently at horizontal orientations. The distribution of preferred orientation of the inhibitory population showed a horizontal bias before learning, with more neurons preferring horizontal orientation (Figure 4D left), as confirmed by the HBI values (t-test between before and during learning, p < 2.2E-16; t-test between before and after learning, p < 2.2 E-16, Figure 4E). Moreover, the activity of the inhibitory population, represented by the population tuning curve, showed higher activity at the horizontal orientation, matching the population activity of excitatory neurons (Figure 4F magenta lines). As learning proceeded, the population tuning curve, as well as the distribution of preferred orientation of inhibitory neurons, became more isotropic, as the horizontal bias disappeared in the excitatory population. We propose that the nonuniform recurrent inhibition provided by inhibitory neurons plays a key role in the emergence of probabilistic representation in the network.

**Figure 5:**
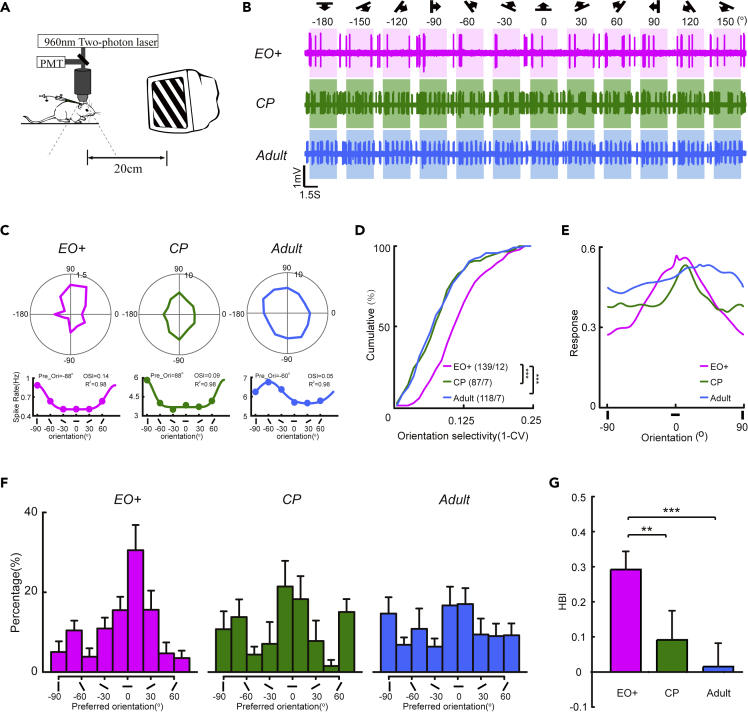
The orientation selectivity and preferred orientation bias of PV + neurons in mouse V1 change at different postnatal stages (A) Schematic illustrating *in vivo* two-photon imaging-guided cell-attached recording (green, Alexa 488) of spike responses from layer ⅔ PV + neurons. (B) The responses of representative neurons at different postnatal stages to drifting gratings with 12 different directions. The stimulus directions and orientations are indicated by the symbols shown above. (C and D) Upper, the orientation-tuned responses of representative neurons in (A) Lower, curves denoting the method to use one-peak Gaussian function fitting to detect the preferred-orientation of representative neurons in (A). (D) Cumulative distributions of OSI values of PV + neurons at the 3 postnatal stages. (E) Population tuning curves of PV + neurons at the 3 postnatal stages. (F) Distributions of preferred orientations of PV + neurons in EO + mice (126 cells/12 mice), CP mice (74 cells/seven mice), and Adult mice (100 cells / 7 mice). (G) HBI of PV + neurons at the 3 postnatal stages. ∗p < 0.05,∗∗p < 0.01, ∗∗∗p < 0.001, Kolmogorov–Smirnov test in D and t-test in (G) Magenta, EO+; green trail, CP; blue trail, Adult. Data are represented as mean ± SEM.

Our model suggests that inhibitory neurons provide nonuniform inhibition at the early learning stage, and this becomes more isotropic at a later stage. Here, we examine whether such a phenomenon exists in PV + inhibitory interneurons, the major players in feedback inhibition in cortical circuits (Hu et al., 2014). We implemented *in vivo* two-photon laser imaging-guided cell-attached recording to measure spiking responses specifically from the PV + interneurons in V1 binocular area, responding to the same drifting gratings with 6 orientations (Figure 5A). To examine the developmental changes, we recorded PV + interneurons in the mice shortly after EO (P17-18), during the CP (P27-28), and in the adult stage (P55-56) (Figure 5B). By calculating the OSI, we found that the orientation tuning of PV + interneurons was stronger shortly after the EO than in the two later stages (Figure 5C). The OSI amplitudes of PV + interneurons decreased significantly from EO to the CP (two-sample Kolmogorov–Smirnov test between P17-18 and P27-28, p = 1.47E-7), and they remained low through the adult stage (Figure 5D, two-sample Kolmogorov– Smirnov test between P17-P18 and P55-56: p = 2.93E-8). These results largely agree with previous experimental findings that the inhibitory PV + interneurons exhibit an initially strong orientation tuning shortly after EO (Kuhlman et al., 2011; Li et al., 2012).

We further analyzed the distribution of preferred orientation of PV + interneuron at different developmental stages. Strikingly, we found that their preferred orientation distributions also showed an apparent bias to the horizontal orientation at the earlier stage and this bias gradually disappeared in development (Figure 5F), a process closely resembling the findings in the PC population (Figure 3). The HBI was highest for PV + interneuron shortly after EO, and decreased during the CP (t-test, p = 0.032), and adult stage (t-test, p = 0.001, Figure 5G). Consistently, the population tuning curves of PV + interneurons showed a horizontal bias shortly after EO, and then disappeared later in development (Figure 5E). Taken together, these experiment results strongly suggest that PV + interneurons provide nonuniform inhibition in the cortical network during early cortical development, endorsing the hypothesis raised by the model simulations.

### Nonuniform feedback inhibition is necessary for the emergence of probabilistic representation

Our model and experiment results suggested that inhibitory neurons provide nonuniform feedback inhibition during development, but how this contributes to visual development is not known. We hypothesized that the nonuniform feedback inhibition is necessary for the emergence of probabilistic representation in the cortical network for the following reasons: first, different from WTA inhibition, feedback inhibition provides a softer form of inhibition in the cortical network (Avermann et al., 2012; Hofer et al., 2011), where the level of inhibition is proportional to the level of excitation (Isaacson and Scanziani, 2011). This softer form of inhibition allows a subset of excitatory neurons to respond to a specific input feature; this is ideal for representing a probability distribution in the network. Second, when applied with the STDP learning rule, the feedback inhibition induces excitatory neurons to compete for the right to respond to input patterns, enabling a powerful learning paradigm referred to as ”competitive learning” (Grossberg, 1976; Masquelier et al., 2009; Nessler et al., 2013). As a result, the distribution of preferred stimuli has an appropriate shape with respect to the input prior probabilities: if the prior probability of an input pattern is higher, a larger fraction of neurons will prefer that pattern; however, if an input pattern is overrepresented in the network, the level of inhibition will rise and prevent excitatory neurons from learning that pattern. In other words, the network can be described by an adaptive k-WTA computation (Lange, 1992), where k is adaptively determined by the prior probabilities and the level of inhibition in the network.

**Figure 6:**
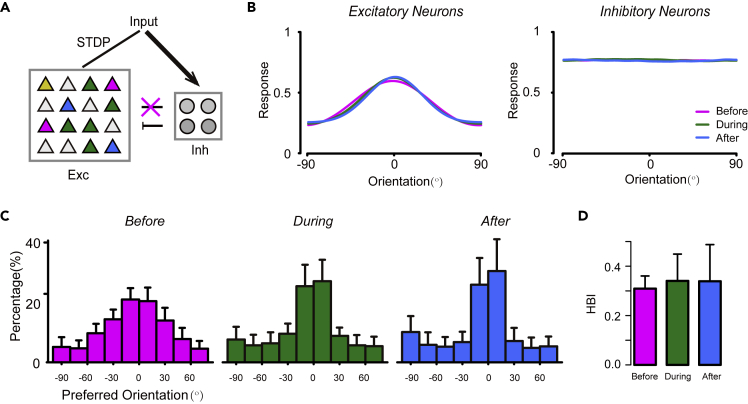
Failed emergence of probabilistic representation in the absence of competitive inhibition (A) Schematic of the network structure with ablation of connections from cortical excitatory neurons to inhibitory neurons. (B) Population tuning curves of excitatory (left) and inhibitory (right) populations at different learning stages (magenta, green, and blue lines indicate before, during, and after learning). (C) Distribution of preferred orientation of excitatory neurons before (left), during (middle), and after (right) learning. (D) HBI of simulated excitatory neurons at different learning stages. Data are represented as mean ± SEM.

To test our hypothesis, we replaced the nonuniform feedback inhibition in the network to a uniform feedforward inhibition, by ablating the recurrent connections from excitatory neurons to inhibitory neurons (Figure 6A). We also increased the feedforward connection weight received by the inhibitory population to keep their mean firing rates unchanged. As a result, the inhibition in the network was provided directly by the feedforward input, and was independent from the activity of the excitatory population (Figure 6B). Under these conditions, the excitatory population failed to transform from a horizontal bias to a cardinal bias (Figure 6C). Our results suggest that nonuniform feedback inhibition, rather than uniform feedforward inhibition, is necessary for the emergence of probabilistic representation in the cortical network.

### Analysis using a simplified rate model

Our theoretical and experimental results suggested that the prior probability and nonuniform inhibition are essential to generate probabilistic representation in the cortical network. To further demonstrate this point, we constructed a simplified rate model to elucidate the effects of the prior probability and nonuniform inhibition on the dynamics of synaptic plasticity.

**Figure 7:**
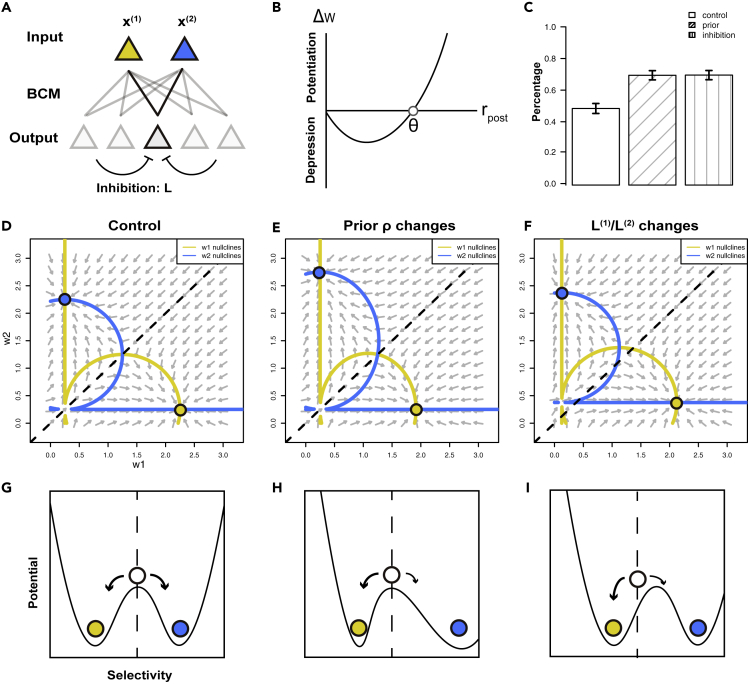
Analysis using a simplified rate model (A) Schematic of the simplified rate model. Input units encode two patterns, presented with different prior probabilities and indicated by yellow and blue. Mutually inhibiting output units receive feedforward connections, which are modified by the BCM rule. (B) In the BCM learning rule, the postsynaptic activity has a nonlinear effect on the change of connection weights, stabilized by a dynamic threshold. (C) Percentage of output units preferring pattern 1 after training under three different conditions: control group, higher prior probability of pattern 1, and decreased inhibition ratio. (D–F) Phase planes of the feedforward synaptic weight for the control group (D), when the prior probability of pattern one increases (E) or the inhibition ratio decreases (F). (G–I) Schematics of the potentials governing the dynamics of the synaptic weights for the groups in (D–F). Yellow and blue circles represent attractor states that are selective for pattern 1 and 2, and the white circle represents the nonselective state. Data are represented as mean ± SEM.

In the simplified model, we consider a network of N mutually inhibiting neurons with linear dynamics, and they receive input patterns x with synaptic weights $w_{i}=\left\{w_{1i},w_{2i}\right\}$ (Figure 7A). The feedforward synaptic weights were modified according to a homeostatic rate-dependent learning rule, namely the Bienenstock-Cooper-Munro (BCM) rule (Bienenstock et al., 1982), which is directly related to the STDP rule (Izhikevich and Desai, 2003):

(Equation 1) $$\tau_{w}\frac{\mathrm{d}w_{ki}}{\mathrm{d}t}=x_{k}y_{i}\left(y_{i}-\theta_{i}\right)\text{,}$$

In the BCM rule, the activity of postsynaptic neuron $y_{i}$ has a nonlinear effect on the changes of synaptic weights $w_{ki}$, whereas the effect of presynaptic neuron $x_{k}$ is linear, with $\theta_{i}$ as a ”sliding threshold” (Figure 7B). In our case, we consider two input patterns with different prior distributions, i.e., $P\left(x^{\left(1\right)}\right)=\rho$ and $P\left(x^{\left(2\right)}\right)=1-\rho$. The effect of nonuniform inhibition was captured by the ratio of the inhibition level under different input patterns ($L^{\left(1\right)}/L^{\left(2\right)}$), which was influenced by the proportions of neurons preferring different patterns. Previous studies have analyzed how orientation selectivity emerges in the network (Cooper and Scofield, 1988; Intrator and Cooper, 1992; Udeigwe et al., 2017), but phase plane analysis has not been applied and the effect of prior probabilities and nonuniform inhibition on the probabilistic representation of the network has not been explored.

We focused on inspecting the synaptic weights of a nonselective neuron, while keeping the weights of other neurons fixed (by setting their weights near to the attractors). This implies that the ratio of inhibition levels under different input patterns ($L^{\left(1\right)}/L^{\left(2\right)}$) are held constant during learning. First, we studied the dynamics of synaptic weights under three conditions: (1) uniform prior and uniform inhibition: ρ=0.5 and $L^{\left(1\right)}/L^{\left(2\right)}=1$, (2) nonuniform prior and uniform inhibition ρ=0.6 and $L^{\left(1\right)}/L^{\left(2\right)}=1$, and (3) uniform prior and nonuniform inhibition: ρ=0.5 and $L^{\left(1\right)}/L^{\left(2\right)}=1/2$. Under uniform prior and uniform inhibition, the neuron had equal chances to become selective to either pattern (Figure 7C). But under nonuniform prior or nonuniform inhibition, the chances were no longer equal: the neuron had a higher chance of becoming selective to the pattern with the higher prior or the lower level of inhibition (Figure 7B). To further unveil the effects of nonuniform prior and feedback inhibition on the network dynamics, we performed phase plane analysis on the synaptic weights from different input units. For uniform prior and uniform inhibition, the phase plane was symmetrical to the diagonal line (Figure 7D), indicating that the neuron had equal chances to become selective to either pattern. But when the prior probability became nonuniform, e.g., ρ increased, the attractor that was selective to pattern $x^{\left(1\right)}$ became closer to the diagonal line, whereas the attractor to $x^{\left(2\right)}$ moved away (Figure 7E). In such a case, the neuron has a higher chance to become selective to $x^{\left(1\right)}$. When the feedback inhibition became nonuniform, e.g., $L^{\left(1\right)}/L^{\left(2\right)}$ decreased, the basin boundary separating the two basins of attraction shifts toward the upper-left, similar to the effect of increasing the prior probability of pattern 1 (Figure 7F). In summary, our analysis showed that nonuniform prior and feedback inhibition shape the changes of synaptic weights, and this affects the preference of neurons in the network (Figures 7G–7I).

### The learned internal representation supports Bayesian-like inference

Learning and inference are not separable in the brain (Friston, 2003; Fiser et al., 2010). However, the validation methods of previous biologically-plausible learning models solely concentrated on comparing the “receptive field” properties of model units with those of neurons. In addition, how the learned model contributes to inference is left implicit (for exceptions, please see Rao and Ballard (1999); Nessler et al. (2013)). Here, we show that our network is able to perform Bayesian-like inference based on the learned internal representation, where the posterior of the orientation can be readout by a population vector decoder (Georgopoulos et al., 1986; Ferger et al., 2021).

To decode the orientation from neural activity, we constructed an encoder-decoder model based on the network described in the previous section (Girshick et al., 2011; Fischer and Peña, 2011). The network encoded the ground truth orientation θ in the population activity, from whom an estimate of the orientation $\hat{\theta }$ was decoded by a population vector decoder (Figure S1A). The population vector decoder computed the sum of the directional vectors associated with the preferred orientation for each neuron, weighted by its firing rates (Figures S1B). Previous studies have shown that if the distribution of preferred stimuli matches the prior probabilities of the stimuli, and the tuning curves of neurons follow Gaussian shapes, the population vector will be consistent with the Bayesian estimate of the stimulus (Fischer and Peña, 2011; Girshick et al., 2011; Ganguli and Simoncelli, 2014). Because the distribution of preferred orientation shifted from a horizontal bias to a cardinal bias during development (Figure S1C), we expected the decoded orientations to have different Bayesian characteristics in terms of estimation variability and bias across different developmental stages.

The variability of the decoded orientation was computed as the *SD* of the estimated orientation given the ground-truth orientation, which is relevant to the discrimination thresholds in psychophysics (Green and Swets, 1966). Before learning, the variability was lowest at horizontal orientation and highest at vertical orientation, suggesting that the discriminability was best at horizontal orientations and worst at vertical orientations for young mice (Figure S1D, left). However, after learning, the variability was lowest at cardinal orientations and highest at oblique orientations. Thus, the results suggest that the discriminability in adult mice is best at cardinal orientations and worst at oblique orientations (Figure S1D, right), consistent with the “oblique effect” reported in monkeys and humans performing orientation discrimination tasks (Appelle, 1972; Bauer et al., 1979). In general, the discriminability was the best at the peak of the distribution of preferred orientation of the encoding population. Recent research has shown that mice are able to discriminate between horizontal and vertical orientations (Long et al., 2015; You and Mysore, 2020), but their discriminant thresholds for different orientations have not been tested systematically.

The nonuniform distribution of preferred orientations in the encoding population causes a bias in estimation (Girshick et al., 2011; Wei and Stocker, 2015). The perceptual bias was computed as the expected difference between the estimated and ground-truth orientations, conditioned on the ground-truth orientation. In our model, the bias showed a “prior attraction” effect, where the prior distribution led to an attraction shift of the estimator (Figure S1B) (Girshick et al., 2011). In the network before learning, the perceptual bias was unimodal, indicating that orientations were perceived to be closer to the horizontal (Figure S1E, left). In addition, the bias was 0 at horizontal and vertical cardinal orientations, and as large as 12° at oblique orientations. For the network after learning, the perceptual bias became bimodal, indicating that orientations are perceived to be oriented closer to the nearest cardinal orientation (Figure S1E, right). The bias was 0 at cardinal and oblique orientations, and as large as 10° in between. A recent experiment has shown that neuronal representation in V1 of adult mice affects their behavioral bias (Jin and Glickfeld, 2019). In summary, the cortical network supports Bayesian inference based on the learned representation.

## Discussion

In this work, we combined mathematical modeling with electrophysiological experiments to study the emergence of probabilistic representation in the cortical network of mouse V1 during development. We found that PV + inhibitory neurons played an important role in the emergence of such a representation through feedback inhibition. With electrophysiology recording, we found a novel and tight relationship between the orientation tuning of PCs and PV + inhibitory neurons across different developmental stages. Furthermore, an ablation study of the network model showed that the nonuniform feedback inhibition is necessary for the emergence of probabilistic representation. Lastly, we show that the network supports Bayesian-like inference based on the learned internal representation. In summary, our results support the proposal that the microcircuits consisting of PCs and PV + inhibitory interneurons are the building blocks for Bayesian inference (Darlington et al., 2018).

The orientation-tuning properties of the PV + inhibitory interneurons changed structurally during visual development, and this was suggested to be crucial for sharpening the orientation selectivity and initiating the CP of excitatory binocular plasticity in the developing visual cortex (Kuhlman et al., 2011; Li et al., 2012). PV + inhibitory interneurons are orientation-selective at early developmental stages (shortly after EO) and become unselective before the onset of the CP. Because the orientation selectivity of PV + inhibitory neuron tends to reflect net biases of the surrounding neurons (Kerlin et al., 2010; Packer and Yuste, 2011), we proposed that such structural changes in PV + inhibitory interneurons reflects shifts in the preferred orientations of the PCs during development. Shortly after EO, the local PC population is biased toward horizontal orientations, thus the PV + neurons have high orientation selectivity and are likely to prefer horizontal orientations (Figure S2A). Then, at the later stage of development, the horizontal bias in PC population is lost, and the number of PCs preferring a specific orientation and its orthogonal orientation is likely to be equal, resulting in a lower orientation selectivity in PV + inhibitory neurons (Figure S2B).

Primary sensory cortex may simultaneously employ different strategies to represent uncertainty. Besides the framework of probabilistic representation that we studied here, where the prior distribution is embedded in the distribution of preferred stimuli (Fischer and Peña, 2011; Girshick et al., 2011; Ganguli and Simoncelli, 2014), theorists have proposed other frameworks for probabilistic representation in primary sensory cortex. Berkes et al. Berkes et al. (2011) proposed that the spontaneous activity of PCs represents the prior distribution of stimuli, whereas the stimulus-evoked activity represents the posterior. Their framework is supported by experimental data in ferret V1, where the spontaneous activities reflect prior expectations of the activities evoked by natural scenes. The probabilistic population code (Ma et al., 2006) proposes that the uncertainty of the stimulus can be encoded by the gain of the population tuning curve. Recent experiments (Walker et al., 2020) suggest that tuning widths of the likelihood function decoded from V1 explain the behavioral variability, and this partly supports the probabilistic population code. But the circuit mechanism for acquiring the above probabilistic representations is not understood and needs to be investigated in future research.

We provide several testable predictions: First, we predict that feedback inhibition is necessary for the emergence of overrepresentation of cardinal orientations during normal visual development. This prediction can be tested by lowering the activity of PV + inhibitory interneurons, possibly by knocking down PV or GAD65 gene expression in V1. Second, we predict that, as a consequence of the nonuniform distribution of preferred orientation in the neural population, the discrimination threshold of mice is nonuniform: lowest at horizontal orientations in mice shortly after EO, and lowest at cardinal orientations in adult mice. This can be directly tested in mice using orientation discrimination tasks (Long et al., 2015; You and Mysore, 2020).

Learning, representation and inference are not separable in the brain. Our computational modeling and *in vivo* electrophysiological recording suggest that the feedback inhibition circuit shapes the emergence of probabilistic representation and supports Bayesian-like inference, which may serve as a building block for Bayesian inference in the brain.

### Limitation of the study

First, although our model suggests feedback inhibition provided by PV + inhibitory neurons are necessary for the emergence of probabilistic representation in the network, we did not test it in experiment by directly manipulating their activity for following reasons: This experiment requires the selective expression of chemogenetic or optogenetic tool proteins in the developing PV + cell, to allow specific manipulation of their activity at a very early developmental stage. A Cre/loxP recombination approach is needed to achieve this (Taniguchi et al., 2011), particularly PV-Cre knock-in mice and Cre-activated adeno-associated viral vectors (AAVs) (Kumar et al., 2020; Boyden et al., 2005; Zhu et al., 2016). However, when the interneuron progenitors migrate tangentially to the cortex and differentiate into different subtypes in this transgenic mouse, PV(/Cre) expression in cortical interneurons does not occur until the second postnatal week (in the PV-Cre mouse) (Chen et al., 2020; van Versendaal and Levelt, 2016; Himmelhan et al., 2018; Ueno et al., 2017). Moreover, stable expression of target genes with AAV vectors in neurons often requires ¿two weeks (Challis et al., 2019). Thus, it is not possible for us to gain stable expression of chemogenetic or optogenetic proteins specifically in developing PV + cells before the third postnatal week. Thus, we are not able to test the role of PV + inhibitory neurons by directly manipulating their activity.

Second, our current research focuses on the developmental role of the major player of feedback inhibition, the PV + inhibitory neurons, but other types of inhibitory neuron may also regulate the emergence of probabilistic representation in V1. Similar to PV + neurons, somatostatin (SOM) inhibitory neurons have dense and reciprocal synaptic connections with nearby PCs (Fino and Yuste, 2011). However, compared to PV + neurons, the inhibition provided by SOM neurons is more facilitatory (Gentet et al., 2012; Miao et al., 2016), more orientation-selective (Ma et al., 2010; Tremblay et al., 2016), and targeted to distal dendrites of PCs. Thus, SOM neurons may regulate the visual development through a more accurate form of inhibition that gates the feedforward inputs received by PCs. Moreover, Vasoactive Intestinal Polypeptide (VIP)-Expressing inhibitory neurons regulate visual development through disinhibition. A recent study showed that developmental dysfunction of VIP interneurons eliminates the bias of PCs toward horizontal stimuli (Batista-Brito et al., 2017). The emergence of probabilistic representation may involve the circuits including various subtypes of inhibitory neurons.

## STAR★Methods

### Key resources table

**Table:** 

REAGENT or RESOURCE	SOURCE	IDENTIFIER
**Experimental models: Organisms/strains**		

Mosue: PvalbIRES-Cre	Jackson Laboratory	Jax No. 008069
Mosue: Rosa26-CAGtdTomato (Ai9)	Jackson Laboratory	Jax No. 007914
Mouse: C57/BL6N	Beijing Vital River Laboratory Animal Technology	213

**Chemicals**		

Alexa Flour 488	Invitrogen	46602

**Apparatus or software**		

Digidata 1440A	Molecular Devices	https://www.moleculardevices.com/
Axon MultiClamp 700B	Molecular Devices	https://www.moleculardevices.com/
pClamp10	Molecular Devices	https://www.moleculardevices.com/
MATLAB 2015b	The MathWorks	https://ch.mathworks.com/
LabView	National Instruments	http://www.elecfans.com/templets/ni2015/
CRT	Sony	G520

### Resource availability

#### Lead contact

Requests for further information regarding resources and reagents used in this study should be directed to the lead contact, Prof. Xiaohui Zhang (xhzhang@bnu.edu.cn).

#### Materials availability

This study did not generate new unique reagents.

### Method details

The circuit model consists of a two-layer neural network. The first layer is the input layer with $N_{P}$ Poisson neurons. The second layer represents the cortical network, which contains $N_{E}$ excitatory and $N_{I}$ inhibitory neurons, mimicking the interacting populations of pyramidal cells and PV + inhibitory neurons in mouse V1.

#### Cortical neurons

The cortical layer is modeled by a recurrent network of N=100 leaky integrate-and-fire neurons, of which 80% are excitatory and 20% are inhibitory. The sub-threshold dynamics of membrane potential $u_{i}$ of neuron *i* follows

(Equation 2) $$\tau_{m}\frac{\text{d}u_{i}}{\mathrm{d}t}=-\left(u_{i}-E_{l}\right)+I_{\text{P},i}+I_{\text{E},i}+I_{\text{I},i}\text{,}$$

where the membrane time constant $\tau_{m}=20$ ms and the resting potential $E_{l}=-70$ mV. $I_{\text{P},i}$ describes the current received from the input layer, $I_{\text{E},i}$ and $I_{\text{I},i}$ are the recurrent inputs from cortical excitatory and inhibitory neurons. When the membrane potential reaches the threshold $u_{th}=-50$ mV, a spike is generated and transmitted to all post-synaptic neurons, then the membrane potential is reset to the resting potential $u_{0}=-70$ mV.

#### Input

The cells in the input layer are modeled as Poisson neurons with time-varying firing rates, which depends on the 1-dimensional stimulus. The continuous orientation pattern θ∈[0,π] is encoded by $N_{P}=100$ Poisson neurons using a positional code with a Gaussian profile, illustrated in Fig.??A. The firing rate of input neuron *i* in response to a stimulus at orientation θ at time t is:

(Equation 3) $$r_{i}\left(t\right)=r_{0}+r_{1}\text{exp}\left(-\frac{{\left(i-\frac{N_{p}}{\pi }\theta \right)}^{2}}{2\sigma^{2}}\right),\sqrt{a^{2}+b^{2}}$$

where $r_{0}$ and $r_{1}$ are the spontaneous and maximum firing rate of the input neuron, σ is the spreads of the Gaussian function (Song and Abbott, 2001). Periodic boundary conditions are imposed to avoid edge effects.

The prior probabilities of the input patterns are described in the simulation detail sections Simulation Details.

#### Network connectivity

The excitatory neurons are reciprocally and randomly connected with inhibitory neurons, with connection probabilities $p_{\text{EI}}=p_{\text{IE}}=50\text{\%.}$ Inbitory neurons are recurrently connected with each other with a probability $p_{\text{II}}=75\text{\%.}$ The recurrent connections between excitatory neurons are not included in the model for simplicity, but simulations with the recurrent connections included did not change our results. The connection probabilities are based on the connectivity data of pyramidal cells with PV^+^ inhibitory neurons in V1 from (Miao et al., 2016). Both excitatory and inhibitory neurons receive feedforward connections from the input layer with probabilities $p_{\text{PE}}=100\text{\%}$ and $p_{\text{PI}}=40\text{\%.}$

#### Synaptic currents

The synapses from input neurons to cortical neurons are all excitatory, with a reversal potential $V_{E}=0\text{ mV,}$ synaptic constant $\tau_{\text{syn}}=3\text{ ms,}$ and synaptic conductance $g_{\text{PE}}$ and $g_{\text{PI}}$ for excitatory and inhibitory populations. The input-cortical synaptic current $I_{\text{P},i}$ received by cortical neuron *i* in neural population A∈{E,I} is

(Equation 4) $$I_{\text{P},i}\left(t\right)=g_{\text{P},i}\left(V_{i}-V_{E}\right),$$

with $g_{\text{P},i}=\left(g_{\text{PA}}/\tau_{syn}\right)\sum_{j=1}^{N_{P}}w_{ji}^{PA}\sum_{k}\text{exp}\left(-\left(t-t_{k,j}\right)/\tau_{\text{syn}}\right)$, where $w_{ji}^{PA}$ is the weight of the input-cortical connection from input neuron *j* to cortical neuron *i*, and $t_{k,j}$ is the time of the *k*-th spike generated by input neuron *j*.

The cortical excitatory and inhibitory synaptic currents have similar forms to those of the input synaptic currents. The cortical excitatory input received by neuron *i* in population A∈{E,I} is

(Equation 5) $$I_{\text{E},\text{i}}\left(t\right)=-g_{\text{E},i}\left(V_{i}-V_{E}\right),$$

with $g_{\text{Ei}}=\left(g_{\text{EA}}/\tau_{syn}\right)\sum_{j=1}^{N_{E}}w_{ji}^{EA}\sum_{k}\text{exp}\left(-\left(t-t_{k,j}\right)/\tau_{syn}\right)$, and the synaptic conductance $g_{\text{EI}}=0.06$.

The inhibitory input received by neuron *i* in population A∈{E,I} is

(Equation 6) $$I_{\text{I},\text{iA}}\left(t\right)=-g_{\text{Ii}}\left(V_{i}-V_{I}\right),$$

with $g_{\text{Ii}}=\left(g_{\text{IA}}/\tau_{syn}\right)\sum_{j=1}^{N_{I}}w_{ji}^{IA}\sum_{k}\text{exp}\left(-\left(t-t_{k,j}\right)/\tau_{syn}\right)$, where synaptic conductance $g_{\text{IE}}=0.02$ and $g_{\text{II}}=0.02$

### Plasticity rule

The connection weights from input neurons to excitatory neurons are plastic and modified by the standard STDP rule illustrated in Figure 1B (Song and Abbott, 2001). The connection weights $w_{ij}$ are updated as follows. For each post-synaptic spike at time $t_{\text{post}},$ only the nearest-neighbor preceding presynaptic spike is considered. For each such pre-before-post spike pair with time difference $t_{\text{post}}-t_{\text{pre}},$ the weight is potentiated by

(Equation 7) $$\Delta w_{ij}\left(t_{\text{post}}-t_{\text{pre}}\right)=\eta_{+}e^{-\frac{t_{\text{post}}-t_{\text{pre}}}{\tau_{+}}},$$

with time constant $\tau_{+}=14$ ms and learning rate $\eta_{+}=0.005.$ For each presynaptic spike at time $t_{\text{pre}},$ the only nearest-neighbor preceding post-synaptic spike is considered. For each such post-before-pre pair with time difference $t_{\text{pre}}-t_{\text{post}},$ the weight is depressed by

(Equation 8) $$\Delta w_{ij}\left(t_{\text{pre}}-t_{\text{post}}\right)=-\eta_{-}e^{-\frac{t_{\text{pre}}-t_{\text{post}}}{\tau_{-}}},$$

with time constant $\tau_{+}=34$ ms and learning rate $\eta_{-}=0.0022$ (in keeping $\frac{\eta_{-}\tau_{+}}{\eta_{+}\tau_{-}}=1.05$).

To avoid unbounded weight growth, we introduce a fast homeostatic plasticity. We keep the sum of the feedforward weights received by an excitatory neuron $\sum_{j=1}^{N_{P}}w_{ji}^{PE}$ constant at its initial value, by rescaling the weights after each update. Negative or unconnected synaptic weights are set to zero. Weights of non-plastic connections, which remain constant during simulation, are set to 1 if two neurons are connected, and 0 otherwise.

#### Population vector decoder

We use a population vector decoder to decode the orientation from the network response. The population vector is computed as a linear combination of the preferred orientation vectors of the neurons, weighted by the firing rates given the input orientation θ

(Equation 9) $$\text{PV}\left(\theta \right)=\frac{1}{N}\sum_{i}R_{i}\left(\theta \right)u\left(\theta_{i}\right),$$

where $u\left(\theta_{i}\right)$ is a unit vector pointing to the preferred orientation of the $i^{\text{th}}$ neuron’s and $R_{i}\left(\theta \right)$ is the firing rate of the $i^{\text{th}}$ neuron, drawn either from a Poisson or Gaussian distribution as described above. The estimated orientation $\hat{\theta }$ is found by computing the direction of the population vector using the inverse tangent.

#### Simplified rate model

In our simplified rate network model, we consider a network of *N* mutually-inhibiting neurons and *K* input units representing *K* patterns (Cooper and Scofield, 1988; Intrator and Cooper, 1992). The activities of neurons follow linear dynamics:

(Equation 10) $$\tau_{y}\frac{\mathrm{d}y_{i}}{\mathrm{d}t}=-y_{i}+\sum_{k}w_{ki}x_{k}-\gamma \sum_{j\neq i}y_{j}\text{,}$$

where $y_{i}$ is the firing rate of neuron *i*, $x_{k}$ is the activity of input unit *k*, $w_{ki}$ is the synaptic weight from input unit *k* to neuron *i*, γ is the lateral inhibition parameter, and $\tau_{y}$ is the time constant. The synaptic weights evolve according to the BCM rule as (Bienenstock et al., 1982):

(Equation 11) $$\tau_{w}\frac{\mathrm{d}w_{ki}}{\mathrm{d}t}=x_{k}y_{i}\left(y_{i}-\theta_{i}\right)\text{,}$$

(Equation 12) $$\tau_{\theta }\frac{\mathrm{d}\theta_{i}}{\mathrm{d}t}=y_{i}^{2}-\theta_{i},$$

where θ is the “sliding threshold” that models homeostatic processes, and $\tau_{w}$ and $\tau_{\theta }$ are time constants. The system can be further simplified by assuming that the firing rate and homeostatic processes have faster dynamics than the synaptic plasticity $\left(\tau_{y},\tau_{\theta }\ll \tau_{w}\right),$ which gives

(Equation 13) $$y_{i}=\sum_{k}w_{ki}x_{k}-\gamma \sum_{j\neq i}y_{j}\text{,}$$

(Equation 14) $$\tau_{w}\frac{\mathrm{d}w_{ki}}{\mathrm{d}t}=x_{k}y_{i}\left(y_{i}-\theta_{i}\right),$$

(Equation 15) $$\theta_{i}=Ey_{i}^{2}.$$

For illustration purposes, we chose two orthogonal input patterns (*K* = 2), with $x^{\left(1\right)}=\left[1,0\right]$ and $x^{\left(2\right)}=\left[0,1\right]$ (for correlated input patterns, please see Udeigwe et al. (2017)). The probabilities of presenting $x^{\left(1\right)}$ and $x^{\left(2\right)}$ are ρ and 1−ρ. This learning system is stochastic in nature. To gain more insight, we can transform the stochastic dynamic system to a deterministic one using the mean-field approach, by studying the average effect of the probabilistic input on the synaptic weight (Cooper and Scofield, 1988; Intrator and Cooper, 1992; Udeigwe et al., 2017). A mean field equation for the synaptic weights is:

(Equation 16) $$\tau_{w}\frac{\mathrm{d}w_{1i}}{\mathrm{d}t}=\rho y_{i}^{\left(1\right)}\left(y_{i}^{\left(1\right)}-\theta_{i}\right),$$

(Equation 17) $$\tau_{w}\frac{\mathrm{d}w_{2i}}{\mathrm{d}t}=\left(1-\rho \right)y_{i}^{\left(2\right)}\left(y_{i}^{\left(2\right)}-\theta_{i}\right),$$

where $y_{i}^{\left(1\right)}$ and $y_{i}^{\left(2\right)}$ are the firing rates of neuron *i* under input pattern 1 and 2 respectively, and $\theta_{i}=\rho y_{i}^{\left(1\right)2}+\left(1-\rho \right)y_{i}^{\left(2\right)2}.$ Similarly, the mean field equation for the firing rates of neuron *i* as a consequence of synaptic plasticity is:

(Equation 18) $$\tau_{w}\frac{\mathrm{d}y_{i}^{\left(1\right)}}{\mathrm{d}t}=\rho y_{i}^{\left(1\right)}\left(y_{i}^{\left(1\right)}-\theta_{i}\right),k$$

(Equation 19) $$\tau_{w}\frac{\mathrm{d}y_{i}^{\left(2\right)}}{\mathrm{d}t}=\left(1-\rho \right)y_{i}^{\left(2\right)}\left(y_{i}^{\left(2\right)}-\theta_{i}\right).$$

The above equations allow us to analyze the effect of prior probabilities and non-uniform feedback inhibition using phase plane-analysis. By setting the above derivatives to 0, we get the fixed points of the dynamic system: $\left(y_{i}^{\left(1\right)},y_{i}^{\left(2\right)},\theta_{i}\right)=\left\{\left(\frac{1}{\rho },0,\frac{1}{\rho }\right),\left(0,\frac{1}{\rho },\frac{1}{\rho }\right),\left(0,0,0\right),\left(1,1,1\right)\right\}$. The fixed points $\left(\frac{1}{\rho },0,\frac{1}{\rho }\right)$ and $\left(0,\frac{1}{\rho },\frac{1}{\rho }\right)$ are stable, while (0,0,0) and (1,1,1) are neither stable nor selective (Castellani et al., 1999). We can study the effects of prior probabilities and non-uniform feedback inhibition on the dynamics of synaptic weight of neuron *i* by changing ρ and $\sum_{j\neq i}y_{j}^{\left(k\right)}$ respectively.

#### Simulation details

In this section, we provide details of the computer simulations reported in the Results. All simulations were performed in R, with a discretization time step Δt of 1 ms. Each simulation was repeated 25 times. All codes are available at https://github.com/AmazingAng/EmergeProbV1.

#### Simulations of the cortical network under stripe rearing condition

For Figures 1C and 1D, the network receives continuous orientation inputs, mimicking the effect of stripe-rearing, where the mice have restricted visual experience with only one orientation (Kreile et al., 2011). The prior probability of orientation inputs follows a von-Mises distribution with its center at the experienced orientation (−90°C, −45°C, 0°C, or 45°C) and concentration κ=0.5 (see Figure 1C). Before training, the feedforward connection weights of excitatory neurons are set according to a Gaussian profile:

(Equation 20) $$w_{ij}^{0}=w_{0}+w_{1}\text{exp}\left(-\frac{{\left(i-\frac{N_{p}\theta_{j}}{\pi }\right)}^{2}}{2\sigma_{w}^{2}}\right),$$

where $w_{0}$ and $w_{1}$ are the minimum and gain factor of the initial weight, $\sigma_{w}$ is the spread of the Gaussian function, $\theta_{j}$ is the initial preferred orientation of neuron *j* (Song and Abbott, 2001). To mimic the over-representation of cardinal orientations in mouse V1, the initial preferred orientation and $\theta_{j}$ is uniformly and randomly distributed in the range 0 to π. During training, each pattern is selected randomly according to its prior probability, corrupted with white noise with a zero mean and 15° s.d., presented for 100 ms. The training period lasts for 1500 s. The tuning curve properties of excitatory neurons in the network are evaluated before and after learning. The synaptic conductances between inputs to cortical neurons are $g_{\text{PE}}=0.012$ and $g_{\text{PI}}=0.006.$

#### Simulations of the feedback inhibition model

We examined the emergent properties of the cortical network model on an input distribution that mimics the distribution of orientations in natural images. Before training, the distribution of preferred orientations $\theta_{j}$ is initialized following a von-Mises distribution with its center μ=π/2 and concentration κ=0.8, based on the over-representation in horizontal orientations in mouse V1 after EO (Hagihara et al., 2015).

During the training phase, the feedforward connection weights of excitatory neurons are modified by the STDP learning rule. Orientation inputs are drawn randomly according to the local orientation distribution measured in photographs p(θ)=c(2−|sin(2θ)|) illustrated in Figure 2A (Girshick et al., 2011; Wei and Stocker, 2015). To measure the orientation tuning of neurons, during the testing phase, STDP in the network is disabled, and the network is tested with 8 orientation steps, each for 1000 ms (see Figures 2B and 4A). Other conditions are the same for Figure 1.

#### Simulations of the cortical network model without competitive inhibition

We examined whether competitive inhibition provided by inhibitory neurons is necessary for the emergence of probabilistic representation in the network. For this purpose, we ablated the synaptic connection from the cortical excitatory population to the inhibitory population, and increased the synaptic conductance from input neuron to inhibitory neuron $g_{\text{PI}}$ from 0.006 to 0.03 (see Figure 6A). This removed all tuned inputs received by inhibitory neurons, while keeping their mean firing rate intact. Other settings were the same for Figure 2.

#### Simulations of Bayesian-like inference from probabilistic representation of the network

Figure S1 shows that the network performs Bayesian-like inference with a population vector decoder, on the embedded probabilistic representation. Theoretical studies have shown that if the probability distribution of preferred stimuli matches the prior probability of the stimuli, and the tuning curves are proportional to the likelihood function, the population vector will be consistent with the Bayesian estimate of the stimulus (Fischer and Peña, 2011).

The network is presented with orientation patterns with step size 1°. Before presentation to the network, orientation patterns are corrupted with white noise of zero mean and 15° s.d. Each orientation is presented for 5 s. The estimated orientations are decoded by a population vector decoder from the responses of excitatory neurons, using a 200-ms moving window. Then, we computed the perceptual variability Var(θ) and bias b(θ) for a given orientation θ by

(Equation 21) $$\text{Var}\left(\theta \right)=\sqrt{\int {\left(\hat{\theta }-\theta \right)}^{2}p\left(\hat{\theta }|\theta \right)\mathrm{d}\hat{\theta }},$$

and

(Equation 22) $$\text{b}\left(\theta \right)=\int \hat{\theta }p\left(\hat{\theta }|\theta \right)\mathrm{d}\hat{\theta }-\theta .$$

### Experimental model and subject details

#### Mouse strains

The mouse strain Pvalb-IRES-Cre (made by S Arbor at the Friedrich Miescher Institute for Biomedical Research, FMI) were obtained from the Jackson Laboratory and crossed with the reporter strain Rosa26-CAG-tdTomato (Ai9) (Jackson Laboratory) (Chen et al., 2014) to obtain PV-Cre:Ai9 mice. The wild-type and transgenic mice were reared in the animal house on a 12/12 h light/dark cycle. All animal procedures are described in protocols which have been reviewed and approved by the Animal Care Committee of the State Key Laboratory of Cognitive Neuroscience and Learning at Beijing Normal University (IACUC-BNU-NKLCNL-2016-02) See Table S1 for details on age and sex of the mice.

#### Visual stimulation

Visual stimuli were generated by a custom-developed software using LabView (National Instruments) and MatLab (Mathworks), and were presented on a 20-inch cathode ray tube (CRT) monitor (Sony Multiscan G520; 30.5 × 30.5 cm; refresh rate, 60 Hz; maximum luminance, 80cd/m^2^), as described in our previous studies (Chen et al., 2014, 2017; He et al., 2014). The CRT monitor was placed 20 cm away in front of the mouse, subtending 80° ×80° of the visual field. Full-screen drifting sinusoidal gratings in 12 different directions (spatial frequency, 0.02 Hz/degree; temporal frequency, 2 Hz) were used to measure the orientation-tuned spiking responses to stimuli in individual recorded neuron. Each stimulation trial consisted of 1 s of blank screen and 3 s of drifting gratings, repeated for 4 times, in which stimulus orientations and directions were randomized.

#### Animal preparation for *in vivo* electrophysiology

Prior to electrophysiological recording, each animal was anesthetized by intraperitoneal injection of ketamine (50μg/g)/medetomidine (0.6μg/g) and mounted on a custom-built mouse stereotaxic device for recording in the visual circuit. The heart rate and body temperature were monitored for the state of anesthesia, and an additional half dose of anesthetic was given to sustain stable anesthesia if necessary during the surgery or recording. Body temperature was maintained at 37°C by a homeostatically controlled heating pad (RWD Life Science). Eye-drops were applied when necessary to prevent drying during the surgery or recording.

#### *In vivo* extracellular recording

Cell-attached from layer 2/3 PV+ interneurons (with tdTomato red fluorescence) in mouse V1 was achieved by the two-photon laser imaging guided target cell recording (He et al., 2014). A cranial window of 2 × 2 mm was made over the V1 area (Madisen et al., 2010). The dura mater in the window was carefully removed and a glass coverslip was mounted on the craniotomy using dental cement, allowing the imaging of fluorescent V1 cells to the depth of 150-400μm (most within layer 2/3) *in vivo*. The targeted cell recording followed a procedure previously described by us (Chen et al., 2014). Glass micropipettes filled with artificial cerebrospinal fluid (aCSF) solution (in mM: 124 NaCl, 2.5 KCl, 2 MgCl_2_, 2 CaCl_2_, 1.25 NaH_2_PO_4_, 26 NaHCO_3_ and 11 D-glucose (pH 7.35)) containing Alexa Fluor 488 (50μM, Invitrogen) (tip diameter, 2μm and resistance 7–10 MΩ) were advanced to the pia at a 14°angle with a micromanipulator (Sutter, MP-255). After contacting the pia, the pipette was further advanced at 1 mm steps towards tdTomato-expressing cells within layer 2/3 under continuous two-photon imaging guidance. A 960-nm two-photon laser was used to excite Alexa 488 and tdTomato. As the pipette was advanced into the cortical tissue, a pressure of 0.2 psi was applied to the micropipette until it touched the cell membrane, indicated by a large drop in the magnitude of the current induced by a test voltage pulse (5 mV, 100 Hz). The positive pressure was then released and a small amount of suction was immediately applied to form a loose seal with resistance ranging from 30 to 800MΩ. This loose-seal configuration recorded spikes from single PV + cells without rupturing the cell membrane.

The extracellular single-unit recording on the layer 2/3 PCs follows a procedure described in our previous studies (Chen et al., 2014; He et al., 2014), using the glass micropipettes filled with the aCSF (5–7MΩ). Before the recording, a cranial window of 2 × 2 mm was made over the V1 area. In a penetration, only spiking activities with wide width were recorded from the neurons located at the cortical depth ¡ 500μm were acquired.

Signals were recorded with an Axon Multi-Clamp 700B micropipette amplifier (Molecular Devices), filtered at 5 kHz (low pass), digitalized by a Digidata 1440A converter board (Molecular Devices) and finally acquired at 10 kHz with pClamp10 (Molecular Devices) into a computer for further analysis. Spike events were detected and further analyzed with a custom program in MatLab (Mathworks). The baseline spike activity was defined by the average spike number within 1 s before the onset of grating stimuli. Evoked spike rates in response to visual stimuli were measured over the duration of stimulation, and the baseline spike rate was subtracted.

In our recording experiments, about 16 pyramidal neurons or 14 inhibitory PV + cells, in average for each animal, were recorded in 3-4 locations spanning across the mediolateral extension of the binocular zone in a 2 × 2 mm cranial window on the mouse brain. This followed a previous recording method (Gordon and Stryker, 1996), in which the ocular dominance profile of visual cortical neurons was quantified representatively across the mouse V1. We adopted the similar recording procedure for representing the orientation selectivity distribution of the V1 in each mouse. The numbers of WT mice were 6 (3 females +3 males), 5 (2 females +3 males) and 8 (3 females +5 males) for P17-18, P27-28 and P55-56 stages, respectively, and that of PV-Cre mice were 12 (6 females +6 males), 7 (3 females +4 males) and 7 (3 females +4 males), respectively. The numbers of recorded PCs and PV + cells from corresponding mice at each age were indicated in the figure legends as well as the Table S1.

### Quantification and statistical analysis

#### Orientation selectivity

The level of orientation selectivity is quantified as the global orientation selectivity index (OSI):

(Equation 23) $$\text{OSI}=\sqrt{{\left(\sum_{i}R\left(\theta_{i}\right)\text{sin}2\theta_{i}\right)}^{2}+{\left(\sum_{i}R\left(\theta_{i}\right)\text{cos}2\theta_{i}\right)}^{2}}/\sum_{i}R\left(\theta_{i}\right).$$

where $\theta_{i}$ is the orientation of the moving gratings, and $R\left(\theta_{i}\right)$ is the spike rate (with baseline subtracted).

#### Preferred orientation

To determine the preferred orientation of a neuron, a Gaussian function was fitted to the mean spike rates evoked by drifting gratings of 6 different orientations (see Figures 3A and 5A), where the spike rate for each orientation was the average spike rate of the two corresponding opposite directions. Cells with $R^{2}>0.6$ were included for further analysis. The preferred orientation was defined as the angle at which the fitted Gaussian function peaked.

#### Population tuning curve

To determine the sensory-evoked population activity, population tuning curves were computed as follows: First, we normalized the orientation tuning curve for each cell to its maximum spike rate (at the preferred orientation).Then, we averaged the normalized responses to different stimuli across neurons in the population.

#### Horizontal bias index

The horizontal bias index (HBI) was defined following (Hagihara et al., 2015):

(Equation 24) HBI=1−dOri(preferredOrientation,horizontal)/45,

which took a value between −1 (vertical) and 1 (horizontal), where dOri(X, Y)=min(|X−Y|,|180−|X−Y||) for each neuron.

## Data Availability

Data reported in this paper will be shared by the lead contact upon request. Original codes are available at https://github.com/AmazingAng/EmergeProbV1. Any additional information required to reanalyze the data reported in this paper is available from the lead contact upon request

## References

[bib1] Appelle S. (1972). Perception and discrimination as a function of stimulus orientation: the “oblique effect” in man and animals. Psychol. Bull..

[bib2] Avermann M., Tomm C., Mateo C., Gerstner W., Petersen C.C.H. (2012). Microcircuits of excitatory and inhibitory neurons in layer 2/3 of mouse barrel cortex. J. Neurophysiol..

[bib3] Bastos A.M., Usrey W.M., Adams R.A., Mangun G.R., Fries P., Friston K.J. (2012). Canonical microcircuits for predictive coding. Neuron.

[bib4] Batista-Brito R., Vinck M., Ferguson K.A., Chang J.T., Laubender D., Lur G., Mossner J.M., Hernandez V.G., Ramakrishnan C., Deisseroth K. (2017). Developmental dysfunction of vip interneurons impairs cortical circuits. Neuron.

[bib5] Bauer J.A., Owens D.A., Thomas J., Held R. (1979). Monkeys show an oblique effect. Perception.

[bib6] Berkes P., Orbán G., Lengyel M., Fiser J. (2011). Spontaneous cortical activity reveals hallmarks of an optimal internal model of the environment. Science.

[bib7] Bienenstock E.L., Cooper L.N., Munro P.W. (1982). Theory for the development of neuron selectivity: orientation specificity and binocular interaction in visual cortex. J. Neurosci..

[bib8] Boyden E.S., Zhang F., Bamberg E., Nagel G., Deisseroth K. (2005). Millisecond-timescale, genetically targeted optical control of neural activity. Nat. Neurosci..

[bib9] Castellani G., Intrator N., Shouval H., Cooper L. (1999). Solutions of the bcm learning rule in a network of lateral interacting nonlinear neurons. Netw. Comput. Neural Syst..

[bib10] Challis R.C., Kumar S.R., Chan K.Y., Challis C., Beadle K., Jang M.J., Kim H.M., Rajendran P.S., Tompkins J.D., Shivkumar K. (2019). Systemic aav vectors for widespread and targeted gene delivery in rodents. Nat. Protoc..

[bib11] Chapman B., Bonhoeffer T. (1998). Overrepresentation of horizontal and vertical orientation preferences in developing ferret area 17. Proc. Natl. Acad. Sci..

[bib12] Chen B., Xu C., Wang Y., Lin W., Wang Y., Chen L., Cheng H., Xu L., Hu T., Zhao J. (2020). A disinhibitory nigra-parafascicular pathway amplifies seizure in temporal lobe epilepsy. Nat. Commun..

[bib13] Chen G., Zhang Y., Li X., Zhao X., Ye Q., Lin Y., Tao H.W., Rasch M.J., Zhang X. (2017). Distinct inhibitory circuits orchestrate cortical beta and gamma band oscillations. Neuron.

[bib14] Chen X.j., Rasch M.J., Chen G., Ye C.q., Wu S., Zhang X.h. (2014). Binocular input coincidence mediates critical period plasticity in the mouse primary visual cortex. J. Neurosci..

[bib15] Cooper L.N., Scofield C.L. (1988). Mean-field theory of a neural network. Proc. Natl. Acad. Sci. U S A.

[bib16] Darlington T.R., Beck J.M., Lisberger S.G. (2018). Neural implementation of bayesian inference in a sensorimotor behavior. Nat. Neurosci..

[bib17] Espinosa J.S., Stryker M.P. (2012). Development and plasticity of the primary visual cortex. Neuron.

[bib18] Ferger R., Shadron K., Fischer B.J., Peña J.L. (2021). Barn owl’s auditory space map activity matching conditions for a population vector readout to drive adaptive sound localizing behavior. J. Neurosci..

[bib19] Fino E., Yuste R. (2011). Dense inhibitory connectivity in neocortex. Neuron.

[bib20] Fischer B.J., Peña J.L. (2011). Owl’s behavior and neural representation predicted by bayesian inference. Nat. Neurosci..

[bib21] Fiser J., Berkes P., Orbán G., Lengyel M. (2010). Statistically optimal perception and learning: from behavior to neural representations. Trends Cognitive Sciences.

[bib22] Friston K. (2003). Learning and inference in the brain. Neural Networks.

[bib23] Furmanski C.S., Engel S.A. (2000). An oblique effect in human primary visual cortex. Nat. Neurosci..

[bib24] Ganguli D., Simoncelli E.P. (2014). Efficient sensory encoding and bayesian inference with heterogeneous neural populations. Neural Comput..

[bib25] Gentet L.J., Kremer Y., Taniguchi H., Huang Z.J., Staiger J.F., Petersen C.C. (2012). Unique functional properties of somatostatin-expressing gabaergic neurons in mouse barrel cortex. Nat. Neurosci..

[bib26] Georgopoulos A.P., Schwartz A.B., Kettner R.E. (1986). Neuronal population coding of movement direction. Science.

[bib27] Girshick A.R., Landy M.S., Simoncelli E.P. (2011). Cardinal rules: visual orientation perception reflects knowledge of environmental statistics. Nat. Neurosci..

[bib28] Gordon J.A., Stryker M.P. (1996). Experience-dependent plasticity of binocular responses in the primary visual cortex of the mouse. J. Neurosci..

[bib29] Green D.M., Swets J.A. (1966).

[bib30] Grossberg S. (1976). Adaptive pattern classification and universal recoding: I. parallel development and coding of neural feature detectors. Biol. cybernetics.

[bib31] Hagihara K.M., Murakami T., Yoshida T., Tagawa Y., Ohki K. (2015). Neuronal activity is not required for the initial formation and maturation of visual selectivity. Nat. Neurosci..

[bib32] He L.j., Liu N., Cheng T.l., Chen X.j., Li Y.d., Shu Y.s., Qiu Z.l., Zhang X.h. (2014). Conditional deletion of mecp2 in parvalbumin-expressing gabaergic cells results in the absence of critical period plasticity. Nat. Commun..

[bib33] Himmelhan D., Rawashdeh O., Oelschläger H. (2018). Early postnatal development of the visual cortex in mice with retinal degeneration. Mech. Dev..

[bib34] Hofer S.B., Ko H., Pichler B., Vogelstein J., Ros H., Zeng H., Lein E., Lesica N.A., Mrsic-Flogel T.D. (2011). Differential connectivity and response dynamics of excitatory and inhibitory neurons in visual cortex. Nat. Neurosci..

[bib35] Hoy J.L., Niell C.M. (2015). Layer-specific refinement of visual cortex function after eye opening in the awake mouse. J. Neurosci..

[bib36] Hu H., Gan J., Jonas P. (2014). Fast-spiking, parvalbumin+ gabaergic interneurons: from cellular design to microcircuit function. Science.

[bib37] Hubel D.H., Wiesel T.N. (1959). Receptive fields of single neurones in the cat’s striate cortex. J. Physiol..

[bib38] Intrator N., Cooper L.N. (1992). Objective function formulation of the bcm theory of visual cortical plasticity: statistical connections, stability conditions. Neural Networks.

[bib39] Isaacson J.S., Scanziani M. (2011). How inhibition shapes cortical activity. Neuron.

[bib40] Izhikevich E.M., Desai N.S. (2003). Relating stdp to bcm. Neural Comput..

[bib41] Jin M., Glickfeld L.L. (2019). Contribution of sensory encoding to measured bias. J. Neurosci..

[bib42] Jonke Z., Legenstein R., Habenschuss S., Maass W. (2017). Feedback inhibition shapes emergent computational properties of cortical microcircuit motifs. J. Neurosci..

[bib43] Kerlin A.M., Andermann M.L., Berezovskii V.K., Reid R.C. (2010). Broadly tuned response properties of diverse inhibitory neuron subtypes in mouse visual cortex. Neuron.

[bib44] Kreile A.K., Bonhoeffer T., Hübener M. (2011). Altered visual experience induces instructive changes of orientation preference in mouse visual cortex. J. Neurosci..

[bib45] Kuhlman S.J., Olivas N.D., Tring E., Ikrar T., Xu X., Trachtenberg J.T. (2013). A disinhibitory microcircuit initiates critical-period plasticity in the visual cortex. Nature.

[bib46] Kuhlman S.J., Tring E., Trachtenberg J.T. (2011). Fast-spiking interneurons have an initial orientation bias that is lost with vision. Nat. Neurosci..

[bib47] Kumar S.R., Miles T.F., Chen X., Brown D., Dobreva T., Huang Q., Ding X., Luo Y., Einarsson P.H., Greenbaum A. (2020). Multiplexed cre-dependent selection yields systemic aavs for targeting distinct brain cell types. Nat. Methods.

[bib48] Lange T.E. (1992). Advances in Neural Information Processing Systems.

[bib49] Lee S.H., Kwan A.C., Zhang S., Phoumthipphavong V., Flannery J.G., Masmanidis S.C., Taniguchi H., Huang Z.J., Zhang F., Boyden E.S. (2012). Activation of specific interneurons improves v1 feature selectivity and visual perception. Nature.

[bib50] Li Y.t., Ma W.p., Li L.y., Ibrahim L.A., Wang S.z., Tao H.W. (2012). Broadening of inhibitory tuning underlies contrast-dependent sharpening of orientation selectivity in mouse visual cortex. J. Neurosci..

[bib51] Long M., Jiang W., Liu D., Yao H. (2015). Contrast-dependent orientation discrimination in the mouse. Scientific Rep..

[bib52] Ma W.J., Beck J.M., Latham P.E., Pouget A. (2006). Bayesian inference with probabilistic population codes. Nat. Neurosci..

[bib53] Ma W.p., Liu B.h., Li Y.t., Huang Z.J., Zhang L.I., Tao H.W. (2010). Visual representations by cortical somatostatin inhibitory neurons—selective but with weak and delayed responses. J. Neurosci..

[bib54] Madisen L., Zwingman T.A., Sunkin S.M., Oh S.W., Zariwala H.A., Gu H., Ng L.L., Palmiter R.D., Hawrylycz M.J., Jones A.R. (2010). A robust and high-throughput cre reporting and characterization system for the whole mouse brain. Nat. Neurosci..

[bib55] Magueresse C.L., Monyer H. (2013). Review gabaergic interneurons shape the functional maturation of the cortex. Neuron.

[bib56] Masquelier T., Guyonneau R., Thorpe S.J. (2009). Competitive stdp-based spike pattern learning. Neural Comput..

[bib57] McAdams C.J., Maunsell J.H. (1999). Effects of attention on orientation-tuning functions of single neurons in macaque cortical area v4. J. Neurosci..

[bib58] Miao Q., Yao L., Rasch M.J., Ye Q., Li X., Zhang X. (2016). Selective maturation of temporal dynamics of intracortical excitatory transmission at the critical period onset. Cell Rep..

[bib59] Nessler B., Pfeiffer M., Buesing L., Maass W. (2013). Bayesian computation emerges in generic cortical microcircuits through spike-timing-dependent plasticity. Plos Comput. Biol..

[bib60] Packer A.M., Yuste R. (2011). Dense, unspecific connectivity of neocortical parvalbumin-positive interneurons: a canonical microcircuit for inhibition?. J. Neurosci..

[bib61] Pouget A., Dayan P., Zemel R. (2000). Information processing with population codes. Nat. Rev. Neurosci..

[bib62] Rao R.P., Ballard D.H. (1999). Predictive coding in the visual cortex: a functional interpretation of some extra-classical receptive-field effects. Nat. Neurosci..

[bib63] Rumelhart D.E., Zipser D. (1985). Feature discovery by competitive learning. Cogn. Sci..

[bib64] Runyan C.A., Sur M. (2013). Response selectivity is correlated to dendritic structure in parvalbumin-expressing inhibitory neurons in visual cortex. J. Neurosci..

[bib65] Sadeh S., Clopath C., Rotter S. (2015). Emergence of functional specificity in balanced networks with synaptic plasticity. PLoS Comput. Biol..

[bib66] Seriès P., Seitz A.R. (2013). Learning what to expect (in visual perception). Front. Hum. Neurosci..

[bib67] Song S., Abbott L.F. (2001). Cortical development and remapping through spike timing-dependent plasticity. Neuron.

[bib68] Stryker M.P., Sherk H., Leventhal A.G., Hirsch H.V. (1978). Physiological consequences for the cat’s visual cortex of effectively restricting early visual experience with oriented contours. J. Neurophysiol..

[bib69] Taniguchi H., He M., Wu P., Kim S., Paik R., Sugino K., Kvitsani D., Fu Y., Lu J., Lin Y. (2011). A resource of cre driver lines for genetic targeting of gabaergic neurons in cerebral cortex. Neuron.

[bib70] Tremblay R., Lee S., Rudy B. (2016). Gabaergic interneurons in the neocortex: from cellular properties to circuits. Neuron.

[bib71] Udeigwe L.C., Munro P.W., Ermentrout G.B. (2017). Emergent dynamical properties of the bcm learning rule. J. Math. Neurosci..

[bib72] Ueno H., Suemitsu S., Okamoto M., Matsumoto Y., Ishihara T. (2017). Parvalbumin neurons and perineuronal nets in the mouse prefrontal cortex. Neuroscience.

[bib73] van Versendaal D., Levelt C.N. (2016). Inhibitory interneurons in visual cortical plasticity. Cell Mol. Life Sci..

[bib74] Walker E.Y., Cotton R.J., Ma W.J., Tolias A.S. (2020). A neural basis of probabilistic computation in visual cortex. Nat. Neurosci..

[bib75] Wei X.X., Stocker A.A. (2015). A bayesian observer model constrained by efficient coding can explain “anti-bayesian” percepts. Nat. Neurosci..

[bib76] You W.K., Mysore S.P. (2020). Visual psychophysics and limits of visual discrimination performance in freely behaving mice. bioRxiv.

[bib77] Zhu H., Aryal D.K., Olsen R.H., Urban D.J., Swearingen A., Forbes S., Roth B.L., Hochgeschwender U. (2016). Cre-dependent dreadd (designer receptors exclusively activated by designer drugs) mice. Genesis.

