# Supplementary material for: Emergence of probabilistic representation in the neural network of primary visual cortex
Source: iScience. 2022 Feb 26;25(3):103975. doi: 10.1016/j.isci.2022.103975 (PMC8924637; doi:10.1016/j.isci.2022.103975)
Supplement: Document S1. Figures S1 and S2 and Table S1 [file mmc1.pdf]

iScience, Volume *25*

Supplemental information

Emergence of probabilistic representation in the neural network of primary visual cortex

Ang A. Li, Fengchao Wang, Si Wu, and Xiaohui Zhang

Supplemental Material

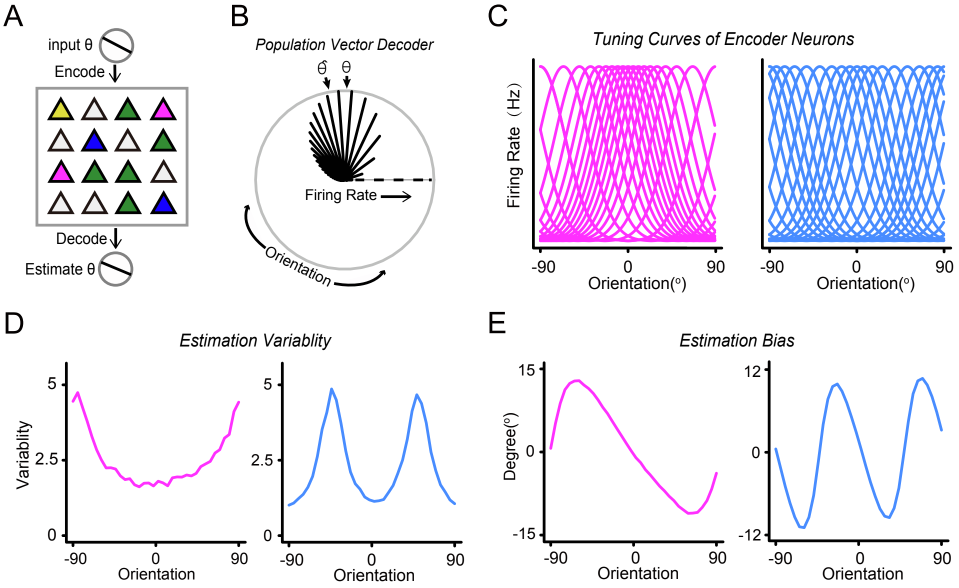

Figure S1. Bayesian-like inference from the probabilistic representation of the network. **Related to Fig.2.**

A. Schematic of the neural encoder-decoder model. Orientation input is encoded by the neural network with neural response. Then the orientation is estimated by the population vector decoder.

B. Schematic of the population vector decoder with a non-uniform neural population.

C. Tuning curves of the encoder neural population with non-uniform orientation preferences before (left) and after learning (right) (the same neural population as in Fig.2; only a subset of neurons is shown).

D. Variability of the estimated orientations before (left) and after learning (right).

E. Bias of the estimated orientations before (left) and after learning (right).}

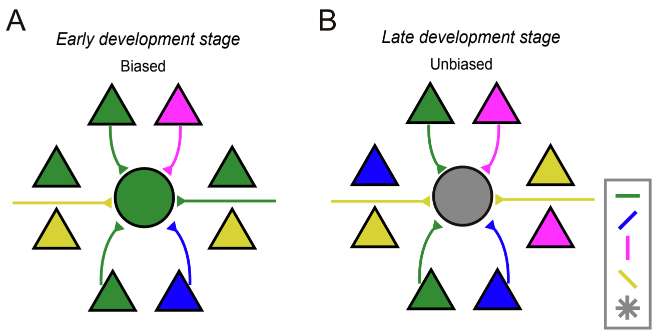

Figure S2. Developmental changes in orientation selectivity of PV+ interneurons reflects the developmental changes in the distribution of preferred orientations local PCs. **Related to Fig.5.**

A. Shortly after EO, the local PC population is biased towards horizontal orientations, thus the PV+ neurons have high orientation selectivity and are likely to prefer horizontal orientations.

B. Before CP, the horizontal bias in the PC population is lost, and the number of PCs preferring a specific orientation and its orthogonal orientation are likely to be equal, resulting in low orientation selectivity in PV+ inhibitory neurons

Age	Number of recorded PCs (WT)	Number of recorded PV+ (PV Cre-Ai9)
P17-18	105 (3 Female + 3 Male)	139 (6 Female + 6 Male)
P27-28	93 (2 Female + 3 Male)	87 (3 Female + 4 Male)
P55-56	117 (3 Female + 5 Male)	118 (3 Female + 4 Male)

Table S1: The Numbers of Recorded Cells, Related to STAR Methods. Pyramidal cells (PCs) and inhibitory parvalbumin-expressing (PV+) interneurons cells from corresponding mice at different postnatal age are listed. Corresponding sexes used for recording are indicated in brackets.
